# Effects of exogenous salicylic acid and pH on pathogenicity of biotrophy-associated secreted protein 1 (BAS1)-overexpressing strain, *Magnaporthe oryzae*

**DOI:** 10.1007/s11356-018-2532-y

**Published:** 2018-06-21

**Authors:** Jing Yang, Yunfeng Wang, Lin Liu, Lina Liu, Chunmei Wang, Changmi Wang, Chengyun Li

**Affiliations:** State Key Laboratory for Conservation and Utilization of Bio-Resources in Yunnan, Yunnan Agricultural University, Kunming, Yunnan China

**Keywords:** *Magnaporthe oryzae*, Rice, Salicylic acid, pH, Pathogenesis-related genes, Abiotic stress

## Abstract

Abiotic stress can influence the interactions between a pathogen and its host. In this paper, we analyzed the effects of salicylic acid (SA) and pH on the morphological development and pathogenicity of *Magnaporthe oryzae*, the pathogen that causes rice (*Oryza sativa*) blast. A strain of rice blast that overexpresses biotrophy-associated secreted protein 1 (BAS1) and a wild-type (WT) strain were pretreated with different levels of pH and different concentrations of SA to analyze *M*. *oryzae* colony growth, sporulation, spore germination, dry weight of hypha, and appressorium formation. Disease incidence and the expression of defense-related genes in infected rice were analyzed after pretreatment with pH 5.00 or pH 8.00 and 200 μM SA. The results showed that both SA and pH had some influence on morphological development, including sporulation and appressorium formation of the BAS1-overexpression strain. In the 200 μM SA pretreatment, there was a lower incidence of disease and higher expression levels of the rice defense-related genes *PR1a*, *PAL*, *HSP90*, and *PR5* on leaves inoculated with the BAS1-overexpession strain compared with the WT strain, whereas, *LOX2* appeared to be downregulated in the BAS1-overexpession strain compared with the WT. In both pH treatments, disease incidence and expression of *HSP90* were higher and the expression of *PR1a* and *PR10a* and *LOX2* and *PAL* was lower in leaves inoculated with the BAS1-overexpression strain compared with leaves inoculated with the WT strain. We conclude that SA and pH affect morphological development of the BAS1-overexpression blast strain, but that these factors have little influence on the pathogenicity of the strain, indicating that BAS1-overexpression may have enhanced the tolerance of this rice blast strain to abiotic stressors. This work suggests new molecular mechanisms that exogenous SA and pH affect the interactions between *M. oryzae* and rice.

## Introduction

The effector proteins secreted by plant pathogens play a key role in the interaction between the pathogens and the host plant as well as in the progression of the disease. Effector proteins are considered to manipulate the host’s cell structure and function, facilitate infection, and suppress the host’s immune response (Kamoun 2006; Hogenhout et al. 2009; Cooper et al. 2016). Once effector proteins enter the host, they can both work in the extracellular matrix and change the host’s cellular environment to facilitate infection and colonization (Kamoun 2006; Ridout et al. 2006; Hogenhout et al. 2009; Białas et al. 2017).

Pathogens secrete various enzymes based on their surrounding environment (Gebauer et al. 2017; Gumtow et al. 2017; Kaverinathan et al. 2017). During the infection period of *Botryis cinerea*, the fungus can secrete various intracellular polygalacturonases to facilitate its infection (Louis et al. 2014). Environmental conditions may also play a role; many studies have reported that temperature and pH can influence the secretion of effector proteins by pathogens. Louis et al. (2014) found different soluble candidate effector proteins were secreted by *Cochliobolus lunatus* at different temperature conditions. Some fungi have evolved complicated regulatory mechanisms to recognize and respond to the surrounding pH changes, such as secreting different proteins according to different pH levels in the surrounding environment (Li et al. 2012). To invade its host successfully, *B. cinerea* can secrete different proteins in different tissues of the host (Sharma et al. 2016). Although some real virulence factors can successfully suppress a host’s defense response, they still need to function in coordination with specific extracellular secreted proteins (Li et al. 2012). The study on *B. cinerea* shows that the culture solution of *B. cinerea* is black under the conditions of pH 4 and pH 6 because of the secondary metabolites produced during the growth of the hyphae (Sharma et al. 2016; Kaverinathan et al. 2017). A separate study showed that the microenvironment (pH) of the host plant could regulate an arsenal of enzymes to increase fungal pathogenicity (Alkan et al. 2013). Thus, we can conclude that pH is a major environmental factor that affects the fungus secreting effector proteins and promotes its colonization in the host’s tissues. Other studies have shown that the *C. lunatus* isolates with higher melanin levels are more virulent than those with lower melanin levels; thus, melanin and related secondary metabolite are considered to be virulence factors of *C. lunatus* (Xu et al. 2007; Gao et al. 2012).

*M*. *oryzae* (Ascomycotina) cause the fungal disease rice blast, which has serious effects on rice (*Oryza sativa*) production, severely impairing rice yield and quality. Under normal conditions, conidia that fall on the surface of the host plant start to germinate. Germ tubes quickly differentiate to form specialized infected cells, i.e., the appressoria. *M. oryzae* have a highly specialized infection structure, an appressorium, which penetrates the host plant (Howard et al. 1991; Jong et al. 1997). After penetrating the host, the invasive hyphae grow rapidly in the host cells and, concurrently, spindle-shape lesions appear. After 5 to 7 days, a large number of conidia are produced on lesions and a new round of the infection cycle begins. During infection, the microenvironment of the host plant cells can change, including the pH, temperature, and humidity around the infection site. The host cells may also increase or decrease levels of hormones, such as salicylic acid (SA) and jasmonate acid (JA), which can affect signal transduction and the production of lysates and secondary metabolites. For example, the expression of the *PR1* gene can be induced by SA and JA (Mitsuhara et al. 2008).

There are also many reports about how the signal transduction pathway in fungal cells is related to pH level. Fungi can grow and develop in a wide range of pH levels because fungi can regulate their surrounding pH by secreting acidic or alkaline substances (Piccirillo et al. 2010; Pen et al. 2011; Landraud et al. 2013); these secretions can further facilitate its ability to infect and colonize a host plant. Thus, environmental factors (e.g., temperature, humidity, pH, active oxygen, and SA) are predicted to play an important role in the interaction between the *M. oryzae* and its rice host. Therefore, understanding the effects of the environmental factors on the interaction between the *M. oryzae* and the host could help to reveal new pathogenic mechanisms of *M. oryzae*.

This paper studied the effects of an exogenous hormone (SA) and pH on the sporulation, spore germination, appressorium formation, and mycelial growth, pathogenicity of a strain of *M. oryzae* that overexpresses biotrophy-associated secreted protein 1 (BAS1). This study aims to provide a platform for further analyses of the effect of abiotic stress on the interaction between the pathogen and its host plant, and provide important theoretical and experimental proofs for guiding the agricultural management.

## Materials and methods

### Materials

The wild-type (WT) strain of *M. oryzae* (95234I-1b) used here is stored in our laboratory. Strain 35S:BAS1/Mo-2 is a transformed strain that overexpresses BAS1; it is also stored in our laboratory and was obtained by transforming the expression vectors of BAS1 and mCherry fusion under the 35S promoter constructed.

Lijiangxintuanheigu (LTH) was used in this paper; the rice variety was a universally susceptible variety, which is also stored in our laboratory.

### Methods

#### Culture of blast strain in media containing different concentrations of SA and different pH values

Mycelial pellets of rice blast strain were placed on a PDA (potato dextrose agar) medium, and then put in a 28 °C incubator for 7 days. The fresh mycelia pellets in PDA were put into a triangular flask filled with potato dextrose broth (PDB) culture medium, and then put the triangular flask in a shaker of 28 °C and 120 rpm for 3 days of culture. We dissolved the SA (Sigma, MO, USA) in a small amount of dimethyl sulfoxide (DMSO) and prepared the culture media for the different SA concentrations. The control (CK) treatment consisted only of the quantity of DMSO required for preparing the highest SA concentration and an aliquot of sterile water. The six SA treatments were as follows: 50 μM, 100 μM, 200 μM, 500 μM, 1 mM, and 2 mM. The pH was adjusted for each culture media (PDA, PDB), by adding 20 mmol/L MES buffer solution or 20 mmol/L MOPS buffer solution and then further adjusted using NaOH or HCl (Landraud et al. 2013).

#### Effects of SA concentration and pH on the growth of *M. oryzae* mycelium

We used mycelia pellets with a puncher of 7 mm in diameter and inoculated them onto PDA media containing different SA concentrations or different pH values. Pellets were placed in the 28 °C incubator for culture. We measured the colony diameters on day 10.

#### Spore production of *M. oryzae*

We inoculated 300 μL *M. oryzae* culture solution into Ximeizhi media containing different SA concentrations and different pH levels and then placed them in a 28 °C incubator for culture for 4 days in the dark, followed by alternate light and darkness (12:12, dark:light) for 6 days. We washed the cultured *M. oryzae* spores with 5 mL sterile water and filtered them with gauze. The filtered solution was mixed uniformly and the spores were counted with a blood-counting chamber.

#### Observation of spore germination and appressorium formation in *M. oryzae*

We adjusted the sterile water containing buffer solution into an aqueous solution of different pH values with NaOH and HCl. We collected the spores of *M. oryzae* from Ximeizhi media. SA solution was added to adjust the spore concentration to 1 × 10^5^ spores/mL. We then took 10 μL of the spore suspension and added it to a hydrophobic slide, and placed the slide in a 28 °C constant-temperature petri dish. We observed spore germination after 2 h and appressorium formation after 6 h.

#### Culture and inoculation of rice seedlings

Rice seeds were sterilized for about 1 min in hypochlorite before sowing. Seeds of LTH were soaked in sterilized water and placed into a 28 °C constant-temperature incubator until dehiscent, and then sown into a seedling bed. Rice seedlings were used for the inoculation experiments when they had formed three leaves (approximately 20 days). To prepare the *M. oryzae* inoculum, we washed the *M. oryzae* spores with sterile water, centrifuged them for about 1 min and discarded the supernatant. Next, we added sterile aqueous solutions of pH 5.00, pH 8.00, or CK (sterile water), and SA solutions of 200 μM with CK (DMSO solution required in 200 μM SA), to adjust the spore suspension to a concentration of 1 × 10^5^ spores/mL, and added in 0.02% Tween 20. The inoculant was applied as a spray to the entire seedlings. Seedlings were kept in the inoculation hood for 24 h after inoculation to preserve the heat and humidity in the darkness after which they were transferred to the greenhouse. About 15 leaves were taken for quantitative analysis 0, 24, 48, 72, 96, and 120 h after the spray inoculation. Seven days after inoculation, we performed a disease investigation. Disease incidence rate was calculated as the number of infected leaves divided by the total number of leaves.

#### Total RNA extraction, cDNA reverse transcription, and qRT-PCR

Total RNA was extracted with RNA extraction kit: Eastepr®Super Total RNA Extraction Kit LS1040 (Promega, WI, USA), following the manufacturer’s instructions. Complementary DNA (cDNA) reverse transcription was done with a reverse transcription kit: GoScriptTM Reverse Transcription System A5001 (Promega), following the manufacturer’s instructions. For quantitative real-time PCR (qRT-PCR) of the target gene, we used primer designs modified from the literature (Marce et al. 2010). The primer sequence is shown in Table 1; actin gene was control. Preparation of 20 μL qRT-PCR reaction system: cDNA of 4 μL, RNA-free water of 5.2 μL, forward and reverse primer of 0.4 μL, respectively, SYBR Premix Ex Taq II (Takara, Tokyo, Japan) of 10 μL. The qRT-PC reaction conditions were as follows: (1) denaturation at 95 °C for 3 min; (2) denaturation at 95 °C for 20 s; (3) annealing and extension at 60 °C for 20 s; (4) collection of fluorescence signal at 65 °C; cycle number of 44. After the cycling, temperature was adjusted from 60 to 98 °C to obtain the dissociation curve.

**Table 1:** Primer sequences of the target genes in rice leaves

Gene	Accession number	Primer pair (5′ → 3′)
*OsPR1a*	Os07g03710	F:5′-GCTACGTGTTTATGCATGTATGG-3′
		R:5′-TCGGATTTATTCTCACCAGCA-3′
*OsPR10a*	Os12g36880	F:5′-AATGAGAGCCGCAGAAATGT-3′
		R:5′-GGCACATAAACACAACCACAA-3′
*OsPAL*	Os02g41680	F:5′-TCACAAGCTCAAGCACCATC-3′
		R:5′-CTCACCAAGCTTCTTGGCAT-3′
*OsEDS1*	Os09g22450	F:5′-AGCTGTGGCAGAAGAGAAGC-3′
		R:5′-GAGCCCCAAAGGTTACACAA-3′
*OsLOX2*	Os02g10120	F:5′-TACAAGTCCGACGAGGAGGT-3′
		R:5′-CCACATGATGGTGGTCAAGA-3′
*OsAOS2*	Os03g12500	F:5′-GGAGGAAGCTGCTGCAATAC-3′
		R:5′-GTGTCGTACCGGAGGAAGAG-3′
*OsHSP90*	Os09g30412	F:5′-CAAGTCGGACCTCGTCAACA-3′
		R:5′-TCTCAGCAACAAGGTAGGCG-3′
*OsPR5*	Os12g43380	F:5′-CGCTTACCTGTTCCCCGAAG-3′
		R:5′-ATGGGCAGAAGACGACTTGG-3′
*actin*	Os11g06390	F:5′-GAGTATGATGAGTCGGGTCCAG-3′
		R:5′-ACACCAACAATCCCAAACAGAG-3′

### Data analysis method

For the qRT-PCR data processing and statistical analysis, we used the 2^−ΔΔCt^ method (Livak and Schmittgen 2001). Three biological replicates and three technical replicates were included in all experiments testing the effects of SA and pH on the growth of mycelium, sporulation quantity, spore germination and appressorium formation, disease investigation, and qRT-PCR. All data and statistical analyses were performed in SPSS 13.0. A two-way analysis of variance (ANOVA) was carried out, followed by the Duncan’s multiple range tests. Graphs were plotted with Sigmaplot 10.0.

## Results

### Effects of SA concentration and pH level on the morphological development of blast strains

To identify the effects of different concentrations of SA and different pH values on the morphological development of the BAS1-overexpression strain, we treated plants inoculated with different concentrations of SA and different pH values (BAS1-overexpression strain and a WT strain of 95234I-1b treated with the same DMSO or sterilized water as controls).

**Fig. 1:**
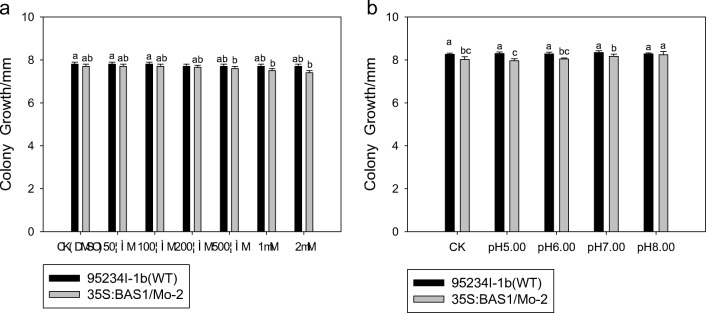
Effect of different concentrations of salicylic acid (**a**) and pH (**b**) on the colony growth of two rice blast strains (*Magnaporthe oryzae*). Values show the means ± SD of three biological replicates. Different letters represent significant difference at *P* ≤ 0.05 using Duncan’s multiple range test

The results showed that there was no significant difference between the different concentrations of SA on the colony growth of the BAS1-overexpressing strain or the WT strain, compared with the control plants of each strain that were pretreated with only DMSO (Fig. 1). This result indicated that SA had no effect on the colony growth of either blast strain.

Colony growth of the BAS1-overexpression strain was lower in the pH 5.00 medium than in the control; however, the difference was not significant. BAS1-overexpression strain cultured in pH 8.00 medium had a significantly higher colony growth than that of the control (Fig. 1). Overall, pH highly affects the BAS1-overexpression strain comparing to the WT strain.

**Fig. 2:**
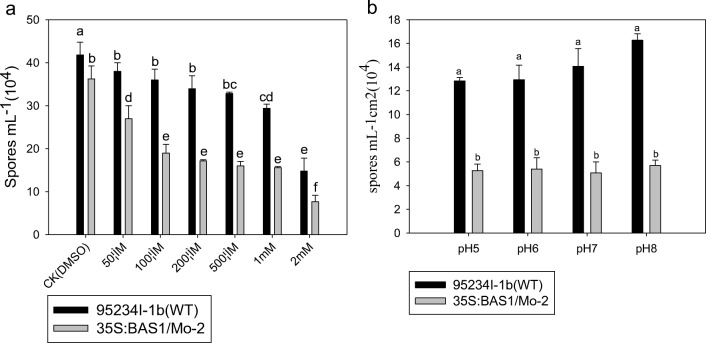
Effect of salicylic acid concentration and pH on number of spores in wild-type (WT) and BAS1-overexpression rice blast strains (*Magnaporthe oryzae*). Values show the means ± SD of three biological replicates. Different letters represent significant difference at *P* ≤ 0.05

SA suppressed sporulation in both the BAS1-overexpression and WT strains compared with the DMSO control (Fig. 2a). The 50 μM SA treatment resulted in greater suppression of sporulation in the BAS1-overexpression strain than in the WT strain, and the 200 μM SA suppressed the sporulation of both the BAS1-overexpression strain and the WT strain (Fig. 2a). However, pH had no significant effects on the sporulation of the BAS1-overexpression strain or the WT strain. However, with the same pH value, the sporulation of the BAS1-overexpression strain was lower than that of the WT strain (Fig. 2b).

**Fig. 3:**
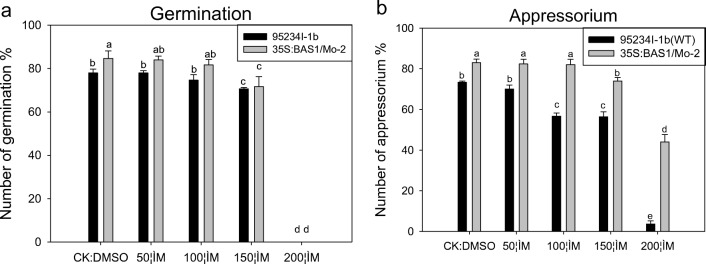
Effects of different SA concentrations on germination (**a**) and appressorium (**b**) of spores in wild-type (WT) and BAS1-overexpression rice blast strains (*Magnaporthe oryzae*). Values show the means ± SD of three biological replicates. Different letters represent significant difference at *P* ≤ 0.05

Two hours after SA treatment, spore germination in the 50 and 100 μM SA treatments was not significantly different than in the CK treatment, whereas the 150 μM SA treatment had significantly less spore germination than CK in both the BAS1-overexpression and WT strains (Fig. 3a). In the 200 μM SA treatment, spore germination was completely suppressed in both blast strains (Fig. 3a). Appressorium formation was observed 6 h after SA treatment. The 50 and 100 μM SA treatments had no suppression of the appressorium formation in either the WT or BAS1-overexpression strains, whereas the 150 μM SA treatment began to suppress appressorium formation of both blast strains and the 200 μM SA treatment completely suppressed appressorium formation in both strains (Fig. 3b). Different concentrations of SA had a greater effect on suppression of appressorium formation in WT compared with the BAS1-overexpression strain.

**Fig. 4:**
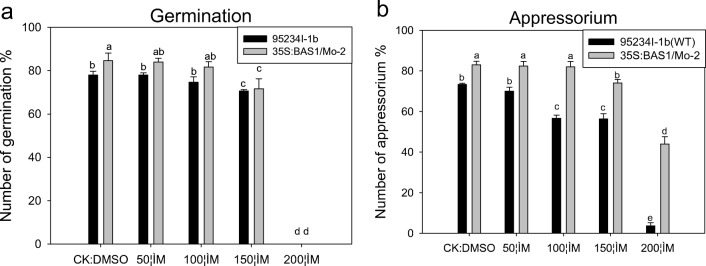
Effects of pH on spore germination and appressorium in wild-type (WT) and BAS1-overexpression rice blast strains (*Magnaporthe oryzae*). Values show the means ± SD of three biological replicates. Different letters represent significant difference at *P* ≤ 0.05

At higher pH values, there was significantly greater suppression of spore germination in the BAS1-overexpression strain compared with the WT strain (Fig. 4a). In contrast, within each strain, different pH values had no significant difference in terms of spore germination rate. The appressorium of BAS1-overexpression strain was observed 2 h after pH treatment, but appressorium was not observed at 2 h after spores of the WT strain were treated with pH 6.00–8.00. Under different pH conditions, the appressorium formation rate was not significantly different for BAS1-overexpression strain, whereas different pH values had an effect on suppression of appressorium formation of WT strain (Fig. 4). The different pH values had no significant effects on the formation rate of appressorium of the BAS1-overexpression strain.

### Effects of different concentrations of SA and different pH values on the pathogenicity of blast strains

We next made an analysis of the pathogenicity of BAS1-overexpression isolates under different concentrations of SA and pH. BAS1-overexpression and WT strains were pretreated with 200 μM SA and pH of 5.00 or 8.00, and disease symptoms were investigated 7 days after the inoculation.

**Fig. 5:**
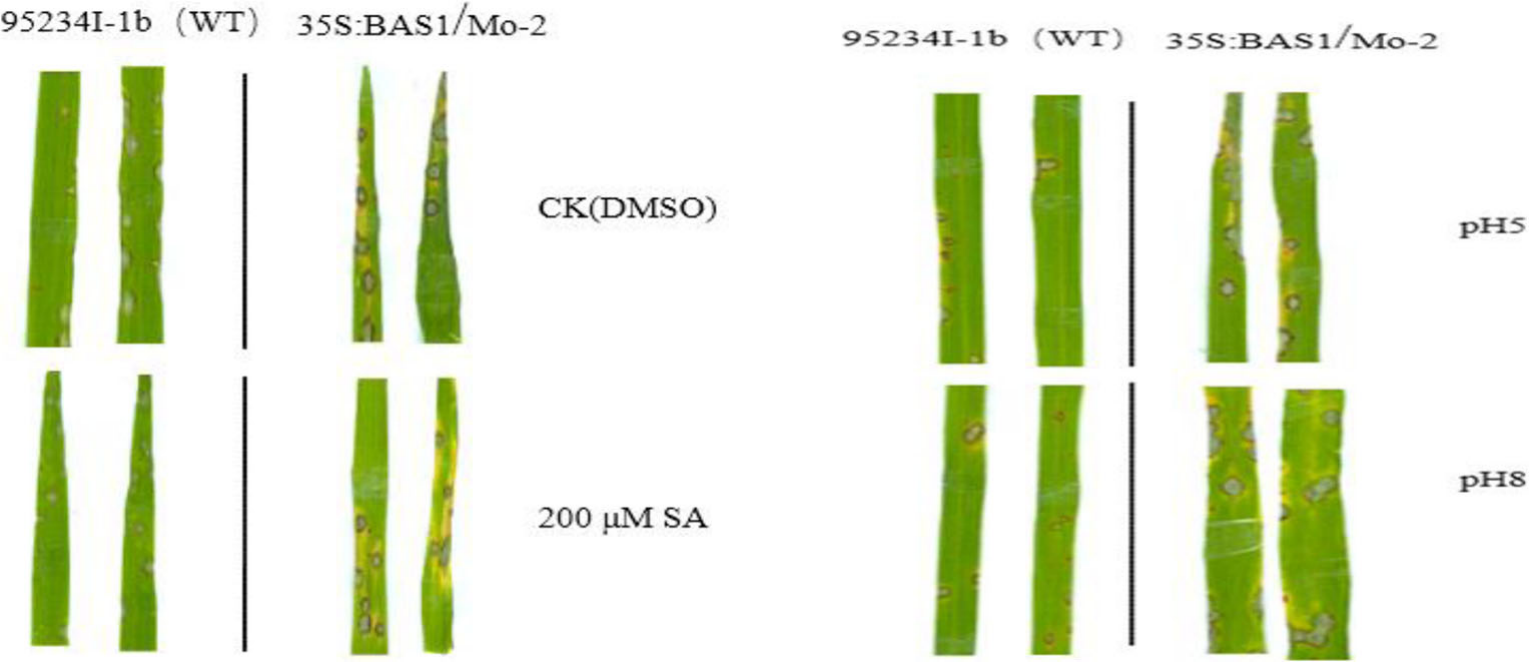
Symptoms on rice leaves pretreated with 200 μM of salicylic acid (**a**) or pH 5.00 or 8.00 (**b**) and then inoculated with a wild-type (WT) or BAS1-overexpression rice blast strain (*Magnaporthe oryzae*)

Disease symptoms were less severe in both blast strains pretreated with 200 μM SA compared with the CK (Fig. 5, Table 2). The results indicated that the disease symptoms in rice seedlings inoculated with spores of BAS1-overexpression strain and WT strain pretreated with 200 μM SA was significantly smaller than that of CK, whereas the decrease of the symptom in rice seedling inoculated with BAS1-overexpression strain pretreated with 200 μM SA was smaller than that of WT strain pretreated with 200 μM SA.

**Table 2:** Disease incidence of spores on rice leaves pretreated with salicylic acid (SA) and inoculated with wild-type (WT) or BAS1-overexpression rice blast strains (*Magnaporthe oryzae*). Values show the means ± SD of three biological replicates. Different letters represent significant difference at *P* ≤ 0.05

Rice leaves	Disease incidence (%)	
	95234I-1b (WT)	35S:BAS1/Mo-2
CK (DMSO)	47.37 ± 4.71b	55.3 ± 5.46a
200 μM SA	32.07 ± 2.03c	41.83 ± 0.85b

The disease symptoms of rice seedlings inoculated with BAS1-overexpression strain pretreated with pH 5.00 and pH 8.00 were more severe than that of WT strain (Fig. 5). The disease incidence rate in rice leaves inoculated with the spore of the BAS1-overexpression strain pretreated with pH 5.00 was 45.67% (Table 3), which is significantly higher than the 36.6% in leaves inoculated with WT strain pretreated with the same pH. We observed a higher disease incidence rate in leaves inoculated with the BAS1-overexpression and WT strains pretreated with pH 8.00 than in leaves inoculated with the WT strain pretreated with pH 5.00 (Table 3).

**Table 3:** Disease incidence in rice leaves pretreated with pH 5.00 or pH 8.00 and inoculated with a wild-type strain (WT) or BAS1-overexpression strain (35S:BAS1/Mo-2) of rice blast. Values show the means ± SD of three biological replicates. Different letters represent significant difference at *P* ≤ 0.05

Rice leaves	Disease incidence (%)	
	95234I-1b (WT)	35S:BAS1/Mo-2
pH 5.00	36.6 ± 5.7b	45.67 ± 2.84a
pH 8.00	43.93 ± 2.29a	46.97 ± 1.32a

### Effects of SA and pH on defense system of rice infected by blast strains

**Fig. 6:**
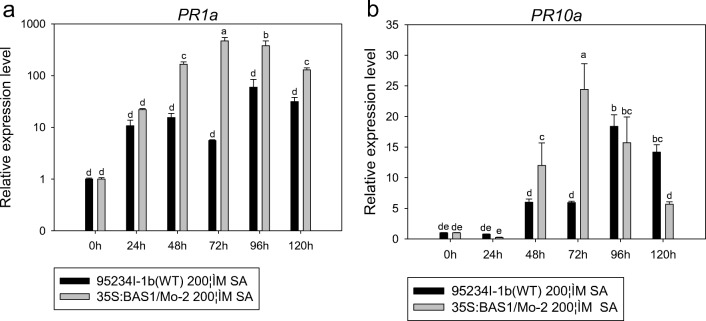
The expression level of *PR1a* and *PR10a* in leaves inoculated with BAS1-overexpression strain pretreated with 200 μM. Values show the means ± SD of three biological replicates. Different letters represent significant difference at *P* ≤ 0.05

We analyzed rice defense-related genes, such as *PR1a* and *PR10a*, *PAL* and *EDS1*, *LOX2* and *AOS2*, and *HSP90* and *PR5* in leaves inoculated with blast strains pretreated with 200 μM SA, pH 5.00 and pH 8.00*. PR1a* in leaves inoculated with BAS1-overexpression strain was higher than in leaves inoculated with WT strain in the 200 μM SA pretreatment (Fig. 6). The expression of *PR1a* in leaves inoculated with the BAS1-overexpression strain pretreated with 200 μM SA was higher at 72 and 96 h post-inoculation (hpi) compared with the other time points studied (24, 48, and 96 hpi), revealing that the expression level of *PR1a* in leaves inoculated with the BAS1-overexpression strain at the later stage of infection was higher than in the earlier stage. The expression level of *PR1a* in leaves inoculated with the BAS1-overexpression strain pretreated with 200 μM SA at 48 and 72 hpi was higher than that of leaves inoculated with the WT strain pretreated with 200 μM SA, whereas the expression level at 96 and 120 hpi was lower than in leaves inoculated with the WT strain pretreated with 200 μM SA (Fig. 6).

**Fig. 7:**
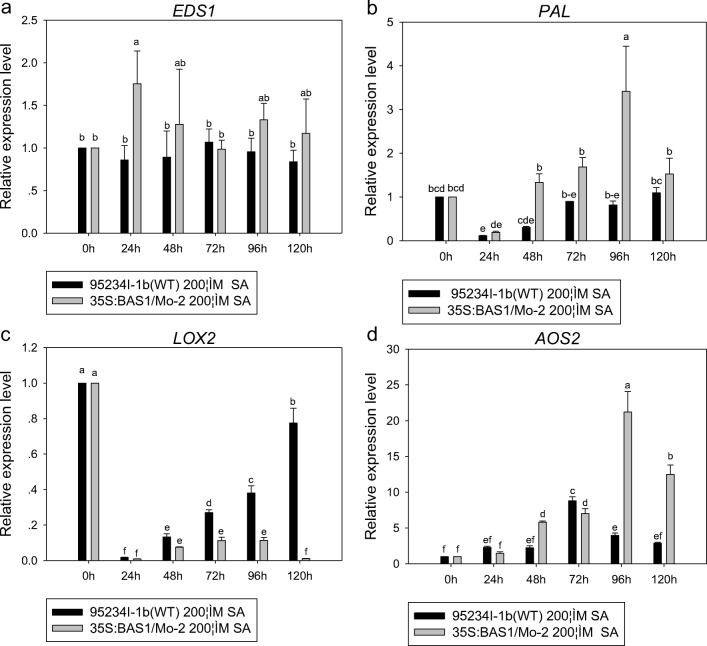
The expression level of *EDS1*, *PAL*, *LOX2*, and *AOS2* in leaves inoculated with BAS1-overexpression strain pretreated with 200 μM. Values show the means ± SD of three biological replicates. Different letters represent significant difference at *P* ≤ 0.05

The expression level of *EDS1* and *PAL* appeared higher in leaves inoculated with BAS1-overexpression strain than in leaves inoculated with the WT strain when both strains were pretreated with 200 μM SA during infection. The highest expression level of *EDS1* and *PAL* inoculated in the BAS1-overexpression strain pretreated with 200 μM SA at 24 and 96 hpi (Fig. 7).

The expression level of *LOX2* in leaves inoculated with the BAS1-overexpression and WT strains pretreated with 200 μM SA appeared downregulated during infection, but there was more downregulation in leaves inoculated with the BAS1-overexpression strain than in leaves inoculated with the WT strain (Fig. 7). The expression level of *AOS2* in leaves inoculated with BAS1-overexpression strain pretreated with 200 μM SA appeared a little upregulation during infection comparing with from 24 to 72 hpi, and it appeared higher expression level of *AOS2* in leaves inoculated with the BAS1-overexpression strain than in leaves inoculated with the WT strain at 96 and 120 hpi (Fig. 7).

**Fig. 8:**
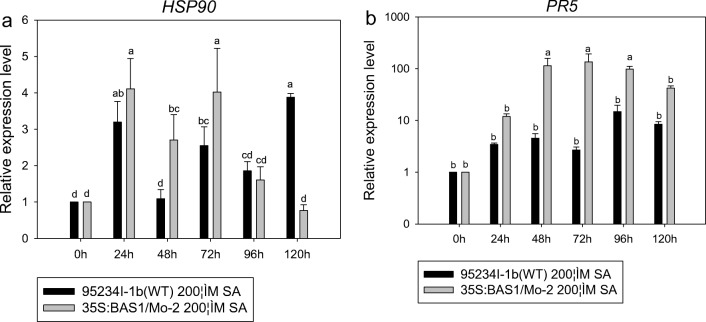
The expression level of *PR5* and *HSP90* in leaves inoculated with BAS1-overexpression strain pretreated with 200 μM. Values show the means ± SD of three biological replicates. Different letters represent significant difference at *P* ≤ 0.05

In the 200 μM SA pretreatments, the expression level of *PR5* in leaves inoculated with the BAS1-overexpression strain was higher than in leaves inoculated with WT strain. The expression level of *HSP90* in leaves inoculated with the BAS1-overexpression strain pretreated with 200 μM SA was significantly higher than that of leaves inoculated with WT strain pretreated with the same SA at 24, 48, and 72 hpi (Fig. 8).

**Fig. 9:**
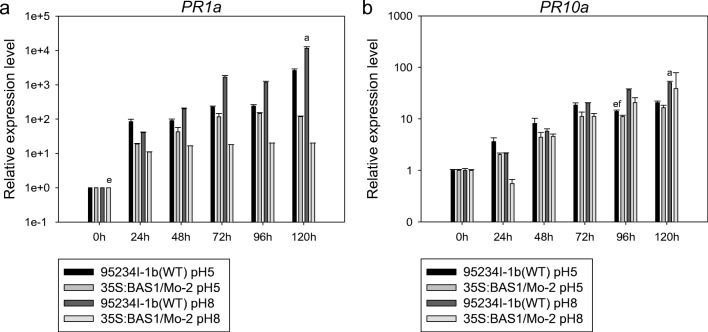
The expression level of *PR1a* and *PR10a* in leaves inoculated with BAS1-overexpression strain pretreated with pH 5.00 and pH 8.00. Values show the means ± SD of three biological replicates. Different letters represent significant difference at *P* ≤ 0.05

The expression levels of *PR1a* and *PR10a* in leaves inoculated with BAS1-overexpression strain and pretreated with pH 5.00 and pH 8.00 were more downregulated than those of leaves inoculated with WT strain pretreated with the two different pH values during infection (Fig. 9). The expression of *PR1a* was lower in leaves inoculated with the BAS1-overexpression strain pretreated with pH 8.00 compared with those pretreated with pH 5.00 (Fig. 9).

**Fig. 10:**
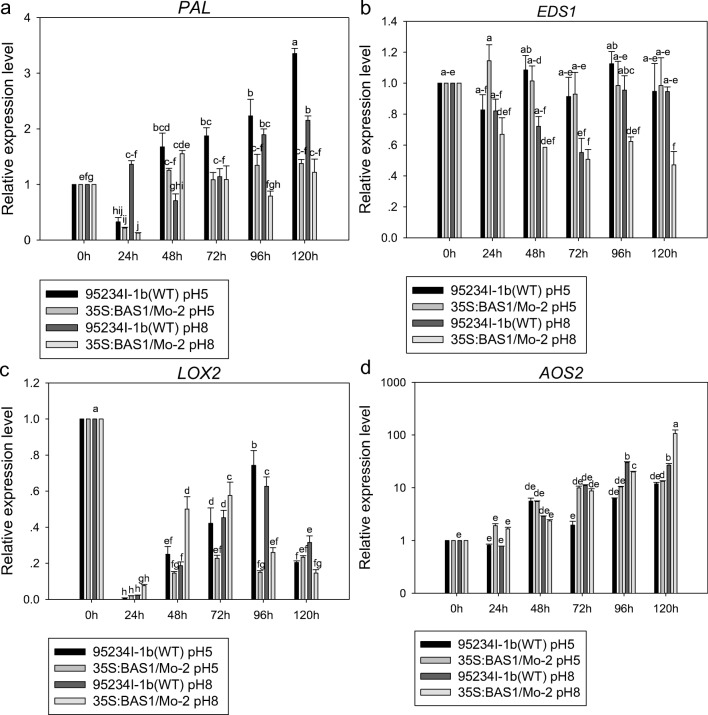
The expression level of *PAL*, *EDS1*, *LOX2*, and *AOS2* in rice leaves inoculated with a BAS1-overexpression strain pretreated with pH 5.00 and pH 8.00. Values show the means ± SD of three biological replicates. Different letters represent significant difference at *P* ≤ 0.05

The expression level of *PAL* appeared lower in leaves inoculated with the BAS1-overexpression strain pretreated with pH 5.00 and pH 8.00 than that of leaves inoculated with WT strain pretreated with the two pH values during infection. Expression levels of *EDS1* appeared lower in leaves inoculated with the BAS1-overexpression strain pretreated with pH 5.00 and pH 8.00 than in leaves inoculated with the WT strain pretreated with the two pH values. There were lower expression levels of *EDS1* and *PAL* in leaves inoculated with the BAS1-overexpression strain pretreated with pH 8.00 than in leaves inoculated with the overexpression strain pretreated with pH 5.00 (Fig. 10).

Expression of *LOX2* in leaves inoculated with the BAS1-overexpression strain and the WT strain pretreated with pH 5.00 and pH 8.00 was significantly downregulated during infection, and lower regulation was observed in leaves inoculated with the BAS1-overexpression strain pretreated with both pH values. The expression quantity of *AOS2* in rice seedlings inoculated by spores of overexpression isolates treated under pH 5.00 and pH 8.00 was significantly upregulated during infection (Fig. 10).

**Fig. 11:**
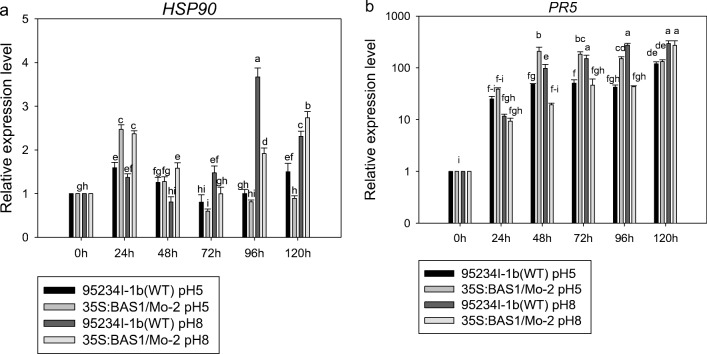
The expression levels of *HSP90* and *PR5* in leaves inoculated with BAS1-overexpression strain pretreated with pH 5.00 and pH 8.00. Values show the means ± SD of three biological replicates. Different letters represent significant difference at *P* ≤ 0.05

The expression level of *HSP90* in leaves inoculated with the BAS1-overexpression strain pretreated with pH 5.00 was significantly upregulated at 24 hpi. The expression level of *HSP90* in leaves inoculated with the BAS1-overexpression strain pretreated with pH 8.00 was higher than in leaves inoculated with the BAS1-overexpression strain pretreated with pH 5.00. The relative expression level of *PR5* in the BAS1-overexpression strain pretreated with pH 5.00 and pH 8.00 was upregulated during infection. However, the relative expression level of *PR5* in leaves inoculated with the WT strain pretreated with pH 5.00 was higher than that of leaves inoculated with BAS1-overexpression strain pretreated with pH 5.00 (Fig. 11).

## Discussion

Climate change could create the environmental conditions for a disease triangle (host plant, pathogen, and environmental conditions suitable for disease development) and this could intensify the occurrence of epidemic plant diseases (Mcelrone et al. 2007). Changes in environment conditions can have a direct impact on pathogens and host plants. During the interaction between *M. oryzae* and rice, *M. oryzae* secretes effector proteins to regulate and manage the cellular structure and physiological metabolism process of rice before infecting the host.

By analyzing the effects of different pH values on the morphological development of blast strains, we found that different pH values had certain effects on the colony growth, spore germination, and other aspects of blast strains; however, it appeared there was no significant effect on the colony growth of WT strains. Landraud et al. (2013) found that the *M. oryzae* strain Guy11 cultured in PDA (potato dextrose agar) medium with pH between 5.00 and 8.00 experienced no significant differences in colony growth. Trushina et al. (2013) also found that the deletion mutant ΔpacC of the *Trichoderma* transcription factor PacC grew at a slow rate in alkaline PDA. Effector proteins have certain acid–base properties, and they can change the acid–base properties of their microenvironment after secreting the effectors, which can promote or suppress the growth of the pathogens. The effect of different pH levels on mycelia growth of several species of dematiaceous fungi (Chai and Liu 2010) and the influence of low pH on arbuscule development, and inhibition effect has been shown to increase (Feng et al. 2017).

We also found that different pH values had an influence on the morphological development of the BAS1-overexpression strain, and that different pH levels could increase the disease incidence rate in rice infected by overexpression isolates. Zou et al. (2010) reported that the function of a few cells is controlled by pH, but the function of these cells was related to pathogen-infecting plants. In this paper, we found some genes appeared upregulated and others downregulated. The expression level of the pathogenesis-related genes *PR1a* and *PR10a* decreased in leaves inoculated with the overexpression strain pretreated with the two pH values. It is well known that the *PR1a* gene is related to the PCD (programmed cell death) signal pathway, and its high expression level promotes the death of a large number of cells in an infected rice seedling and thus suppresses further infection by pathogens (Wu et al. 2014; Fekih et al. 2015). However, in this study, the expression level of *PR1a* decreased, indicating that the death of the rice cells was suppressed, and this facilitated further infection of the overexpression strain. Concurrently, we found that the expression level of *LOX2* and the *PAL* also decreased, indicating that the JA and SA signal pathways of the infected rice seedlings were suppressed, i.e., the defense response of the infected rice seedlings was also suppressed.

Heat shock protein 90 (HSP90) is one of the most broadly studied proteins in HSP families. It functions in cells as a molecular chaperone in response to stress conditions (Yan et al. 2017). We found that the expression level of *HSP90* was upregulated at 24 hpi in leaves inoculated with the overexpression strain and, since *HSP90* is a stress response gene, the upregulated expression of this gene indicated that the infected rice seedlings were under stress response instead of defense response. In the infected rice seedlings, the upregulation of *HSP90* together with the decrease of expression level of *PR1a* promoted the infection and colonization of the overexpression strain.

SA is a metabolite produced in the plant infected by the pathogens by itself and is bound to affect the morphological development of the pathogens to different degrees. SA could induce enzymatic antioxidant activities, related gene expression such as *PR1a* and *PR10a* (Xie et al. 2011; Gill et al. 2016; Kanno et al. 2012), and heat shock protein 90 (HSP90) was one of the most broadly studied proteins in HSP families. It functioned in cells as molecular chaperones in response to stress conditions (Yan et al. 2017). Pathogenesis-related proteins-1 (PR-1), glucanase (Glu), and chitinase (Chi) genes are widely considered is a defensive gene for SA-dependent signaling pathways (Glazebrook 1999).

We found that different concentrations of SA had certain effects on the morphological development of the BAS1-overexpression strain, and we also found a significant decrease in disease incidence rate, and the expression level of *PR1a* and *PR10a* in leaves infected by rice blast strain pretreated with 200 μM SA. These effects appeared to be greatest at the early stages of infection and gradually decrease in the later stages of infection, indicating that SA induces an early defense response in infected rice plants and that the death of rice cells was suppressed at the late stage of infection. *PR1a* is a gene related to the PCD signal pathway, and its expression promotes the death of a large number of cells in an infected rice seedling and thus inhibits further infection of the pathogens (Wu et al. 2014; Fekih et al. 2015). SA plays a key role in the signal transduction path of plant response to biotic stress (Horváth et al. 2007), and the expression of the stress response gene *HSP90* in leaves infected by the overexpression isolates pretreated with 200 μM SA was upregulated at 24 h, indicating that the infected rice seedlings were under significant stress response at the time. In addition, we found that the expression of the *PAL* gene gradually increased over time, indicating that exogenous SA induced the SA signal pathway of the infected rice seedlings. So the BAS1-overexpression strain had higher tolerance to abiotic stress than WT strain. Zhang et al. (2015) found that the expression of the CRN effector protein PsCRN115 of *Phytophthora sojae* in the *Nicotiana benthamiana* improved the resistance of *N. benthamiana* to biotic stress and abiotic stress. Therefore, it can be seen that the expression of pathogen effector proteins in either plants or the pathogens helps improve their tolerance to the external biotic stress and abiotic stress.

## Abbreviations

*AOS2*: Allene oxide synthase 2
BAS1: Biotrophy-associated secreted protein 1
CRN: Crinkling and necrosis induced protein
DMSO: Dimethyl sulfoxide
EDS1: Enhanced disease susceptibility 1
hpi: Hours post-inoculation
*HSP90*: Heat shock protein 90
JA: Jasmonic acid
*LOX2*: Lipoxygenase 2
LTH: Lijiangxintuanheigu
PCD: Programmed cell death
PDA: Potato dextrose agar
PDB: Potato dextrose broth
PsCRN115: Phytophthora sojae crinkling and necrosis induced protein 115
qRT-PCR: Quantitative real-time polymerase chain reaction
*PAL*: Phenylalanine ammonia lyase
*PR1a*: Pathogenesis-related 1a
*PR5*: Pathogenesis-related 5
*PR10a*: Pathogenesis-related 10a
SA: Salicylic acid
WT: Wild-type

## References

[CR1] Alkan N, Espeso EA, Prusk D (2013). Virulence regulation of phytopathogenic fungi by pH. Antioxid Redox Signal.

[CR2] Białas A, Zess EK, De JLC, Franceschetti M, Pennington HG, Yoshida K et al (2017) Lessons in effector and nlr biology of plant-microbe systems. Mol Plant Microbe Interact. MPMI08170196FI 10.1094/MPMI-08-17-0196-FI 29144205

[CR3] Chai B, Liu WD (2010). Effection of different pH on the growth of several species of Dematiaceous fungi. J Trop Med.

[CR4] Cooper B, Campbell KB, Beard HS, Garrett WM, Islam N (2016). Putative rust fungal effector proteins in infected bean and soybean leaves. Phytopathology.

[CR5] Fekih R, Tamiru M, Kanzaki H, Abe A, Yoshida K, Kanzaki E, Saitoh H, Takagi H, Natsume S, Undan JR, Undan J, Terauchi R (2015). The rice (oryza sativa l.) lesion mimic resembling, which encodes an aaa-type atpase, is implicated in defense response. Mol Genet Genomics.

[CR6] Feng ZW, Wang N, Zhu HH, Yao Q (2017). Influences of low pH on the arbuscule development and phosphorus uptake of Rhizophagus intraradices. Mycosystema.

[CR7] Gao S, Liu T, Li Y, Wu Q, Fu K, Chen J (2012). Understanding resistant germplasm-induced virulence variation through analysis of proteomics and suppression subtractive hybridization in a maize pathogen *Curvularia lunata*. Proteomics.

[CR8] Gebauer P, Korn M, Engelsdorf T, Sonnewald U, Koch C, Voll LM. (2017). Sugar Accumulation in Leaves of Arabidopsissweet11/sweet12Double Mutants Enhances Priming of the Salicylic Acid-Mediated Defense Response. Front Plant Sci 8:1378 10.3389/fpls.2017.01378 PMC5550771 28848581

[CR9] Gill RA, Zhang N, Ali B, Farooq MA, Xu J, Gill MB, Mao B, Zhou W (2016). Role of exogenous salicylic acid in regulating physio-morphic and molecular changes under chromium toxicity in black- and yellow- seeded Brassica napus L. Environ Sci Pollut Res.

[CR10] Glazebrook J (1999). Genes controlling expression of defense responses in Arabidopsis. Curr Opin Plant Biol.

[CR11] Gumtow R, Wu D, Uchida J, Tian M (2017). A phytophthora palmivora extracellular cystatin-like protease inhibitor targets papain to contribute to virulence on papaya. Mol Plant-Microbe Interact.

[CR12] Hogenhout SA, Ra VDH, Terauchi R, Kamoun S (2009). Emerging concepts in effector biology of plant-associated organisms. Mol Plant Microbe Interact.

[CR13] Horváth E, Szalai G, Janda T (2007). Induction of abiotic stress tolerance by salicylic acid signaling. J Plant Growth Regul.

[CR14] Howard RJ, Ferrari MA, Roach DH, Money NP (1991). Penetration of hard substrates by a fungus employing enormous turgor pressures. PNAS.

[CR15] Jong JCD, Mccormack BJ, Smirnoff N, Talbot NJ (1997). Glycerol generates turgor in rice blast. Nature.

[CR16] Kamoun S (2006). A catalogue of the effector secretome of plant pathogenic oomycetes. Annu Rev Phytopathol.

[CR17] Kanno H, Hasegawa M, Kodama O (2012). Accumulation of salicylic acid, jasmonic acid and phytoalexins in rice, *Oryza sativa*, infested by the white-backed planthopper*,Sogatella furcifera* (Hemiptera: Delphacidae). Appl Entomol Zool.

[CR18] Kaverinathan K, Scindiya M, Malathi P, Viswanathan R, Sundar AR (2017). Role of melanin in colletotrichum falcatum, pathogenesis causing sugarcane red rot. Sugar Tech.

[CR19] Landraud P, Chuzeville S, Billon-Grande G, Poussereau N, Bruel C (2013). Adaptation to pH and role of PacC in the rice blast fungus *magnaporthe oryzae*. PLoS One.

[CR20] Li B, Wang W, Zong Y, Qin G, Tian S (2012). Exploring pathogenic mechanisms of botrytis cinerea secretome under different ambient ph based on comparative proteomic analysis. J Proteome Res.

[CR21] Livak KJ, Schmittgen TD (2001). Analysis of relative gene expression data using real-time quantitative PCR and the 2 (-Delta Delta C (T) ) Method. Methods.

[CR22] Louis B, Waikhom SD, Roy P, Bhardwaj PK, Singh MW, Goyari S, Sharma CK, Talukdar NC (2014). Secretome weaponries of cochliobolus lunatus interacting with potato leaf at different temperature regimes reveal a cl[xxxx]lhm-motif. BMC Genomics.

[CR23] Marce S, Sawers R, Oakeley E, Angliker H, Paszkowski U (2010). Tissue-adapted invasion strategies of the rice blast fungus magnaporthe oryzae. Plant Cell.

[CR24] Mcelrone AJ, Sherald JL, Forseth IN (2007). Effects of water stress on symptomatology and growth of *Parthenocissus quinquefolia* infected by *Xylella fastidiosa*. Plant Dis.

[CR25] Mitsuhara I, Iwai T, Seo S, Yanagawa Y, Kawahigasi H, Hirose S, Ohkawa Y, Ohashi Y (2008). Characteristic expression of twelve rice PR1 family genes in response to pathogen infection, wounding, and defense-related signal compounds (121/180). Mol Gen Genomics.

[CR26] Pen Y, Chen X, Yang J, Xue M, Wang D, Huang J, Peng Z, Xu J (2011). PacC mediated adaptation to alkaline pH is critical for developing infection hyphae in penetrated plant cells in Magnaporthe oryzae. Phytopathology.

[CR27] Piccirillo S, White MG, Murphy JC, Law DJ, Honigberg SM (2010). The rim101p/pacc pathway and alkaline ph regulate pattern formation in yeast colonies. Genetics.

[CR28] Ridout C, Skamnioti P, Porritt O, Sacristan SJ, Brown J (2006). Multiple avirulence paralogues in cereal powdery mildew fungi may contribute to parasite fitness and defeat of plant resistance. Plant Cell.

[CR29] Sharma M, Sengupta A, Ghosh R, Agarwal G, Tarafdar A, Nagavardhini A, Pande S, Varshney RK (2016). Genome wide transcriptome profiling of fusarium oxysporum f sp. ciceris conidial germination reveals new insights into infection-related genes. Sci Rep.

[CR30] Trushina N, Levin M, Mukherjee PK, Horwitz BA (2013). Pacc and pH–dependent transcriptome of the mycotrophic fungus trichoderma virens. BMC Genomics.

[CR31] Wu L, Chen H, Curtis C, Fu ZQ (2014). Go in for the kill: how plants deploy effector-triggered immunity to combat pathogens. Virulence.

[CR32] Xie X, Xue Y, Zhou J, Zhang B, Chang H, Takano M (2011). Phytochromes regulate SA and JA signaling pathways in rice and are required for developmentally controlled resistance to *Magnaporthe grisea*. Mol Plant.

[CR33] Xu S, Chen J, Liu L, Wang X, Huang X, Zhai Y (2007). Proteomics associated with virulence differentiation of curvularia lunata in maize in China. Acta Bot Sin.

[CR34] Yan J, Liang X, Zhang Y, Li Y, Cao X, Gao J (2017). Cloning of three heat shock protein genes (HSP70, HSP90α and HSP90β) and their expressions in response to thermal stress in loach (Misgurnus anguillicaudatus) fed with different levels of vitamin C. Fish Shellfish Immunol.

[CR35] Zhang M, Ahmed Rajput N, Shen D, Sun P, Zeng W, Liu T, Juma Mafurah J, Dou D (2015). A Phytophthora sojae cytoplasmic effector mediates disease resistance and abiotic stress tolerance in Nicotiana benthamiana. Sci Rep.

[CR36] Zou CG, Tu HH, Liu XY, Tao N, Zhang KQ (2010). PacC in the nematophagous fungus Clonostachys rosea controls virulence to nematodes. Environ Microbiol.

